# A Structural Equation Model of Impulsivity, Psychological Resilience, and Internet Gaming Disorder: Testing Direct and Indirect Pathways Among Saudi University Students

**DOI:** 10.3390/healthcare14131955

**Published:** 2026-07-02

**Authors:** Faihan F. Alshaibany, Majed M. Aljabri, Abdullah M. Alharbi, Bandar S. Alharbi, Alya Alghamdi, Bader M. Almutairy, Waleed M. Alshehri, Norah M. Alyahya, Abdulaziz M. Alodhailah

**Affiliations:** 1 Department of Nursing Administration and Education, College of Nursing, King Saud University, Riyadh 11451, Saudi Arabia; 2 Community and Psychiatric Mental Health Nursing Department, College of Nursing, King Saud University, Riyadh 11451, Saudi Arabia; 3 Department of Medical-Surgical Nursing, College of Nursing, King Saud University, Riyadh 11451, Saudi Arabia

**Keywords:** structural equation modeling, internet gaming disorder, impulsivity, psychological resilience, mediation, measurement invariance

## Abstract

Background: Internet Gaming Disorder (IGD) is a growing mental health concern among university students, often linked to impulsivity and low resilience. However, traditional regression-based research often fails to evaluate latent measurement structures or simultaneously test indirect pathways within a single analytic framework. This study applied structural equation modeling (SEM) to examine the direct and indirect associations between impulsivity, resilience, and IGD symptom severity among Saudi university students, evaluating resilience as a potential mediator and testing measurement equivalence across sex. Methods: Data from 207 students (58.9% male) were analyzed using the lavaan package in R. Confirmatory factor analysis (CFA) evaluated the measurement model for the IGDS9-SF, CD-RISC-10, and S-UPPS-P. Structural paths were tested with indirect effects estimated via bootstrapping (5000 resamples), alongside multi-group invariance testing to examine equivalence across sex. Results: The measurement model demonstrated acceptable fit (CFI = 0.93, TLI = 0.91, RMSEA = 0.058 [90% CI: 0.044, 0.069], SRMR = 0.063). Significant direct associations were observed: impulsivity with IGD severity (β = 0.38, *p* < 0.001) and resilience with IGD severity (β = −0.26, *p* < 0.001). The bootstrapped indirect association of impulsivity with IGD through resilience was statistically significant (β = 0.083, 95% CI [0.031, 0.149]), consistent with partial mediation in this cross-sectional sample. Factor loadings were equivalent across sex (metric invariance supported); the impulsivity–IGD association was stronger in males (β = 0.46) than females (β = 0.27; χ^2^(1) = 4.65, *p* = 0.031), though this comparison should be treated as preliminary. Conclusions: Impulsivity was associated with IGD both directly and indirectly through lower resilience. These SEM-based findings are consistent with resilience as a partial mediator of the impulsivity–IGD association, suggesting potential value in multi-target mental health interventions in university nursing and counseling settings. Longitudinal research is needed to establish causal ordering.

## 1. Introduction

The relationship among impulsivity, psychological resilience, and Internet Gaming Disorder (IGD) has been examined primarily through hierarchical regression and correlation designs [1,2,3]. While these approaches yield important effect size estimates and test specific moderation or main-effect hypotheses, they are constrained in several theoretically important ways. First, they analyze observed scores rather than latent constructs, which conflates true score variance with measurement error and potentially attenuates effect sizes or produces biased estimates of structural relationships [4,5,6]. Furthermore, traditional regression models often fail to account for the unique variance contributed by individual items, leading to a less precise understanding of the underlying psychological phenomena [2,7,8].

A second consideration is that standard regression-based mediation (observed-score path analysis with bootstrapping) can estimate simultaneous direct and indirect effects but does so using observed composite scores rather than latent factors, meaning measurement error is absorbed into the structural estimates rather than modeled separately. Latent-variable SEM separates the measurement and structural layers, providing parameter estimates that more cleanly reflect inter-construct associations under the assumed model [5,9]. Third, many prior studies provide no formal test of measurement equivalence across groups [5,10,11]. This makes cross-sex comparisons potentially misleading if the instruments function differently for male and female respondents, an issue that is particularly relevant given the distinct sociocultural context of Saudi higher education [3,12,13].

Structural equation modeling (SEM) offers several advantages relevant to the present research questions. By estimating latent factors, SEM can reduce the influence of random measurement error on parameter estimates under the assumed model, though it does not eliminate systematic error or confounding. Critically for this study, SEM allows simultaneous estimation of direct and indirect associations within a single model, testing whether the covariance structure is consistent with resilience functioning as a mediator of the impulsivity–IGD association. Additionally, multi-group SEM with invariance testing provides a formal basis for evaluating whether the same construct is being measured equivalently across groups, a prerequisite for meaningful sex comparisons.

The theoretical rationale for hypothesizing a mediated pathway, where impulsivity is associated with lower resilience, which in turn is associated with greater IGD severity, is grounded in both neuropsychological and developmental evidence. Chronically high impulsivity is associated with deficits in executive function, attentional regulation, and emotional modulation [14], which are core components of psychological resilience. Students with high trait impulsivity may, over time, accumulate experiences of emotional dysregulation and coping failures that are associated with lower perceived resilience [15]. Lower resilience, in turn, may be associated with greater reliance on gaming as a coping strategy, consistent with higher IGD symptom severity.

The I-PACE (Interaction of Person-Affect-Cognition-Execution) model explicitly acknowledges that individual difference variables interact through chains of influence rather than operating in isolation [16]. Within this framework, impulsivity is a predisposing person-level factor proposed to shape affective processing and executive regulation, capacities that overlap with what the CD-RISC-10 operationalizes as resilience. The present study tests whether this proposed chain of associations is supported in a Saudi university sample using latent-variable SEM.

Neuroscience-based accounts of prefrontal–striatal circuit function provide additional theoretical context. Within I-PACE, predisposing individual-difference variables, including impulsivity, are proposed to influence affective and cognitive processes that constitute proximal antecedents of addictive behavior. Resilience, understood as a capacity for positive adaptation under stress, can be located within this affective–cognitive regulatory layer. These theoretical propositions motivate the structural model tested in the present study, though no neurobiological variables are directly measured here.

Research indicates that high trait impulsivity is associated with reduced prefrontal cortex (PFC) inhibitory control and heightened striatal reactivity to reward cues [17,18]. Resilience similarly involves PFC-mediated regulation of limbic arousal [19]. These neurobiological accounts provide theoretical grounding for the hypothesized pathway. Most prior studies in this area have examined these constructs as independent predictors without simultaneously modeling the indirect pathway through resilience, particularly in Arab university samples.

The present study addresses these gaps by applying a latent-variable SEM framework to examine direct and indirect associations among impulsivity, resilience, and IGD severity among Saudi university students. The primary objective is to evaluate whether resilience functions as a statistical mediator of the impulsivity–IGD association in this cross-sectional sample, with explicit acknowledgment that the design precludes causal or temporal inference. The model tests three associations: impulsivity with IGD (direct), resilience with IGD (direct), and impulsivity with resilience. Sex-based measurement equivalence is also examined as a secondary objective, given the sociocultural relevance of sex differences in gaming behavior in the Saudi context.

## 2. Materials and Methods

### 2.1. Sample and Instruments

The sample comprised 207 Saudi university students (122 male, 85 female; M_av_^e^ = 21.3 years, SD = 2.6; age range 18–28). Participants were recruited during the 2024–2025 academic year via a stratified cluster convenience sampling procedure across four colleges at King Saud University (College of Nursing, College of Education, College of Sciences, and College of Arts). The four colleges served as the stratification variable. Within each college, classes (clusters) were randomly selected from a list of available second-semester classes provided by the registrar; a convenience element arose because participation was limited to students present on data-collection days. A total of 16 classes were recruited (four per college), each constituting one cluster. Whole classes within randomly selected clusters were invited; all students present on data-collection days were eligible regardless of gaming status. Questionnaires were self-administered in paper form during class time; participation was voluntary, anonymous, and uncompensated. Of approximately 260 questionnaires distributed, all were returned (return rate not separately recorded); 53 were excluded for failing the completeness criterion (>90% item completion per instrument), leaving 207 questionnaires eligible for analysis. This figure (207/260 = 79.6%) represents an effective participation or usable-questionnaire rate rather than a conventional response rate, as the number of questionnaires distributed and eligible students present were approximately equal. All 207 eligible questionnaires were used in the CFA, structural, and multi-group analyses; the MLR estimator handles negligible missing data (<2% across items, no discernible pattern) by full-information maximum likelihood, so no cases were excluded through listwise deletion after the completeness screening. No eligibility criterion restricted participation to active gamers; IGDS9-SF scores therefore reflect self-reported symptom endorsement on a screening instrument and do not constitute a clinical diagnosis of Internet Gaming Disorder. Data collection took place during the second semester of the 2024–2025 academic year (February–April 2025). Power analysis using the MacCallum et al. [20] procedure (null RMSEA ε0 = 0.05, alternative RMSEA ε1 = 0.08, α = 0.05, power = 0.80, model df = 39) indicated a minimum *n* = 188; the obtained sample exceeds this threshold. This df was based on a simplified a priori power model rather than the full measurement model; the reported measurement and structural models have substantially more degrees of freedom (186 and 187, respectively), which means the obtained sample provides adequate power relative to the a priori estimate. The MacCallum procedure is acknowledged as a conservative approach that underestimates power for larger, more constrained models [20]. Validated Arabic versions of all three instruments were administered: the Arabic IGDS9-SF validated by Ali et al. [11] in Arab student populations; the Arabic CD-RISC-10 validated and used in Saudi samples; and the Arabic S-UPPS-P short form adapted and validated in Arab university contexts [13]. These validated translations were used without modification. The three latent constructs were operationalized as follows: the IGDS9-SF (9 items; 5-point Likert scale: 1 = Never to 5 = Very often; total score range 9–45; higher scores indicate greater IGD symptom severity; α = 0.88) [21]; the CD-RISC-10 (10 items; 5-point Likert scale: 0 = Not true at all to 4 = True nearly all the time; total score range 0–40; higher scores reflect greater resilience; α = 0.91) [15,22]; and the S-UPPS-P short form [23] (20 items; 4-point Likert scale: 1 = Agree strongly to 4 = Disagree strongly; items 3, 6, 8, 11, 13, 15, 17, 20 reverse-scored; higher scores indicate greater impulsivity; overall α = 0.82; facet-level α: negative urgency = 0.81, positive urgency = 0.79, lack of premeditation = 0.77, lack of perseverance = 0.76, sensation seeking = 0.80) [24]. Ethical approval was obtained from the Institutional Review Board of King Saud University (reference: 26-0349), and written informed consent was obtained from all participants prior to data collection. All data were stored in password-protected files accessible only to the research team.

### 2.2. Analytic Strategy

All SEM analyses were conducted in R 4.3.1 using the lavaan package (version 0.6-17; Rosseel [25]). The MLR estimator (maximum likelihood with robust standard errors and Satorra–Bentler scaling) in lavaan was applied; Likert-type indicators (4- and 5-point scales) were treated as approximately continuous, which is defensible for five or more response categories but is acknowledged as an assumption. Model comparisons used the Satorra–Bentler scaled chi-square difference test (ΔSBS-χ^2^), as reported in Table 1. Given the sample size (n = 207) and total number of estimated parameters, the 20 S-UPPS-P items were organized into five facet-level parcels (negative urgency, positive urgency, lack of premeditation, lack of perseverance, sensation seeking), each serving as a single indicator of the impulsivity latent factor. Although these parcels correspond to theoretically distinct subdimensions rather than interchangeable items from a unidimensional pool, this approach was adopted as a sample-size-driven modeling convenience following Little et al. [24]; the implications of this choice are discussed in Section 4.6. The IGDS9-SF and CD-RISC-10 items were retained as individual indicators given their lower item counts.

The two-step modeling approach recommended by Anderson and Gerbing [26] was followed. In Step 1, the measurement model was evaluated through CFA, examining standardized factor loadings with 95% CIs, composite reliability (ω), AVE, and HTMT ratios; HTMT values below 0.85 were considered indicative of adequate discriminant validity [27].

Multi-group SEM was used to test measurement and structural equivalence across sex. The invariance testing sequence followed Vandenberg and Lance [28]: configural invariance (same factor structure across groups), metric invariance (equal factor loadings), scalar invariance (equal item intercepts), and structural invariance (equal structural paths). ΔCFI ≤ 0.010 and ΔRMSEA ≤ 0.015 served as criteria for equivalence at each step [29]. Where scalar invariance failed for specific items, partial scalar invariance was estimated by freeing non-equivalent intercepts sequentially guided by modification indices, following Byrne et al. [30]. Because the female subgroup (n = 85) provided limited statistical power for detecting differences in structural paths (estimated power ≈ 49% for a medium effect at α = 0.05) [20], sex-based comparisons should be interpreted as exploratory.

## 3. Results

### 3.1. Descriptive Statistics and Bivariate Associations

Means and standard deviations for primary variables were: IGD severity M = 22.4, SD = 7.8 (range 9–45); resilience M = 25.6, SD = 7.1 (range 3–40); impulsivity M = 46.3, SD = 10.2 (range 24–76). Pearson correlations among latent construct composite scores were: impulsivity–IGD r = 0.47 (*p* < 0.001).

### 3.2. Measurement Model

Table 1 presents CFA fit indices for the three-factor measurement model. The overall chi-square was χ^2^(186) = 312.4, *p* < 0.001) [27]. Composite reliability estimates were 0.89 (IGD), 0.91 (resilience), and 0.84 (impulsivity), each exceeding the 0.70 threshold. Average variance extracted (AVE) values were 0.41 (IGD), 0.53 (resilience), and 0.52 (impulsivity). The IGD AVE of 0.41 falls below the conventional 0.50 threshold, indicating that less than half of the indicator variance is explained by the IGD latent factor, which represents a notable limitation of the IGD measurement model. While composite reliability remained acceptable and the three-factor structure outperformed alternatives in discriminant validity comparisons, the convergent validity of the IGD subscale should be interpreted with caution and acknowledged as a limitation of the present study [27].

Overall measurement model fit was acceptable: CFI = 0.934, TLI = 0.912, RMSEA = 0.057 [90% CI: 0.044, 0.069], SRMR = 0.063. The three-factor model significantly outperformed the most plausible two-factor alternative (ΔSBS-χ^2^(3) = 87.4, *p* < 0.001).

### 3.3. Structural Model Results

The structural model (χ^2^(df = 187) = 315.5, scaling correction factor = 1.15) retained acceptable fit (CFI = 0.928, TLI = 0.906, RMSEA = 0.059 [90% CI: 0.046, 0.072], SRMR = 0.068) and did not differ significantly from the measurement model (ΔSBS-χ^2^(1) = 3.1, *p* = 0.078). The one-degree-of-freedom difference arises because the three-path recursive structural model fixes the three inter-factor correlations to be reproduced exactly by the specified directed paths, imposing one additional constraint relative to the freely correlated CFA in which all three pairwise correlations are estimated freely. A path diagram illustrating standardized coefficients, residual variances, and R^2^ values is provided as Supplementary Figure S1. Standardized and unstandardized direct, indirect, and total effects with confidence intervals are reported in Table 2 and Supplementary Table S2. The direct association from impulsivity to IGD severity was significant and positive (β = 0.38, SE = 0.08, z = 4.75, *p* < 0.001, 95% CI [0.224, 0.536]). The direct association from resilience to IGD severity was significant and negative (β = −0.26, SE = 0.07, z = −3.71, *p* < 0.001, 95% CI [−0.397, −0.123]). The association from impulsivity to resilience was significant and negative (β = −0.32, SE = 0.09, z = −3.56, *p* < 0.001, 95% CI [−0.496, −0.144]), indicating that higher impulsivity was associated with lower resilience in this cross-sectional sample. The model accounted for approximately 10% of the variance in resilience (R^2^ = 0.10, 95% CI [0.03, 0.20]) and 22% of the variance in IGD severity (R^2^ = 0.22, 95% CI [0.12, 0.33]).

### 3.4. Indirect Effect Decomposition

The bootstrapped indirect association of impulsivity with IGD severity through resilience was β = 0.083 (SE = 0.030, 95% CI [0.031, 0.149]), statistically significant by the criterion that the interval excludes zero. The total association of impulsivity with IGD was β = 0.463 (95% CI [0.334, 0.592]); the direct association after accounting for resilience was β = 0.380 (95% CI [0.224, 0.536]). This decomposition is consistent with partial mediation, with resilience accounting for approximately 17.9% of the total association. The proportion mediated is statistically unstable in cross-sectional samples and should not be treated as a precise causal estimate. Alternative causal orderings, including reverse effects of IGD on resilience or influence of unmeasured third variables, cannot be excluded.

### 3.5. Multi-Group Invariance Testing

Table 3 presents invariance test results across sex groups. Configural invariance was established (same factor structure in males and females: CFI = 0.919, RMSEA = 0.063). Metric invariance was supported (ΔCFI = 0.005, ΔRMSEA = 0.003; both within acceptable bounds), indicating that factor loadings are equivalent across sex. Scalar invariance testing revealed two items with significant intercept non-equivalence, IGDS9-SF Item 6 (“Do you feel that you are addicted to internet gaming?”, which reflects perceived addiction rather than behavioral loss of control) and CD-RISC-10 Item 2 (coping effectively under stress)—suggesting that male and female respondents interpret those specific items on somewhat different absolute response scales. Partial scalar invariance was established by freeing these two intercepts; the partially constrained model fit acceptably (ΔCFI = 0.009 from metric model, within bounds).

Table 4 presents the structural path coefficients by sex under the partial scalar invariance model. Under the partial scalar invariance model, path-difference tests were conducted by imposing equality constraints on unstandardized path coefficients across groups. The unstandardized impulsivity–IGD coefficient was significantly larger in males (b = 0.61, SE = 0.13, β = 0.46) than in females (b = 0.36, SE = 0.14, β = 0.27; Δχ^2^(1) = 4.65, *p* = 0.031). Because this comparison was not prespecified, was evaluated alongside two other path tests, and the female subgroup has limited power, it should be treated as exploratory and requires independent replication. The resilience–IGD path did not differ significantly (males: b = −0.38, β = −0.29; females: b = −0.31, β = −0.24; Δχ^2^(1) = 0.68, *p* = 0.412). This pattern is consistent with prior literature reporting stronger impulsivity–IGD associations in males than in females.

## 4. Discussion

This study contributes SEM-based evidence on the associations among impulsivity, psychological resilience, and Internet Gaming Disorder (IGD) in a Gulf Cooperation Council (GCC) university sample, a context underrepresented in the existing literature. By using a latent-variable framework, this research accounts for random measurement error under the assumed model, though it is important to acknowledge that SEM does not resolve problems of confounding, temporal ambiguity, or model misspecification inherent in cross-sectional designs. All findings should be interpreted as associational rather than causal.

### 4.1. Validation of the Measurement Model

Three principal findings emerge from this analysis. First, the measurement model demonstrated that IGDS9-SF, CD-RISC-10, and S-UPPS-P indicators each loaded onto distinct latent factors with acceptable fit, and loadings exceeded the minimum threshold for indicator adequacy, supporting the psychometric functioning of these instruments in a Saudi university context [27]. The three-factor model significantly outperformed both the most plausible two-factor alternative (ΔSBS-χ^2^(3) = 87.4, *p* < 0.001).

### 4.2. The Mediating Role of Resilience

Second, the structural model identified significant direct associations from both impulsivity and resilience to IGD severity, alongside a statistically significant indirect association through resilience. The covariance structure is consistent with resilience functioning as a partial mediator, with the indirect association accounting for approximately 18% of impulsivity’s total association with IGD severity. Because the study is cross-sectional, the direction of these associations cannot be established. Equally plausible alternatives include: IGD severity reducing perceived resilience over time; a shared unmeasured factor (e.g., psychological distress) accounting for associations among all three constructs; or impulsivity partly reflecting a consequence of disordered gaming. These alternatives were not formally tested as sensitivity analyses, which is a limitation.

The 18% mediated proportion warrants cautious interpretation. The proportion mediated is known to be unstable in cross-sectional observational models and should not be treated as a precise estimate of a causal mechanism. The remaining direct association between impulsivity and IGD likely reflects additional pathways not captured in this model, which may include reward-processing tendencies, gaming-specific reinforcement histories, and social or environmental factors. Future longitudinal or experimental studies are needed to test whether these associations reflect causal processes.

### 4.3. Neurobiological and Theoretical Context

These associations are theoretically consistent with neurobiological research, though no neurobiological variables were measured in the present study. The direct impulsivity–IGD association is consistent with neuroimaging findings that individuals with gaming disorder exhibit reduced gray matter volume and altered functional connectivity in the prefrontal cortex (PFC) responsible for inhibitory control [31,32,33]. Trait impulsivity has similarly been associated with prefrontal–striatal connectivity patterns [18], consistent with the theoretical positioning of impulsivity as a predisposing individual-difference variable in the I-PACE model. These neurobiological accounts provide theoretical context for the observed associations, but the present cross-sectional data do not permit neurobiological inferences.

The impulsivity–resilience association is theoretically consistent with developmental accounts in which chronically elevated impulsivity is linked to patterns of regulatory failure that may accumulate as negative experiences and theoretically erode perceived self-efficacy, a process that could manifest as lower resilience scores, though this temporal sequence was not examined in the present cross-sectional data. Students with high trait impulsivity may develop generalized beliefs about their inability to handle stress effectively, reflected in lower resilience scores. These associations are consistent with the I-PACE (Interaction of Person-Affect-Cognition-Execution) model [16], which proposes that predisposing person-level factors influence addictive behaviors through chains of affective-cognitive regulatory processes. Whether these associations reflect the causal sequence described by I-PACE requires longitudinal testing.

### 4.4. Sex Differences and Measurement Invariance

Third, multi-group analysis examined sex-based measurement equivalence. Configural and metric invariance were supported [27,28], confirming equivalent factor loadings across sex and providing a prerequisite for comparing structural paths. Full scalar invariance was not achieved: IGDS9-SF Item 6 (“Do you feel that you are addicted to internet gaming?”; modification index = 14.2) and CD-RISC-10 Item 2 (“I tend to bounce back after illness or hardship”; modification index = 11.8) showed intercept non-equivalence; partial scalar invariance was established by freeing those two intercepts. This constrains latent mean comparisons but allows structural path comparisons. The intercept differences may reflect sex-differentiated response thresholds; acknowledgment of gaming-related loss of control may carry different social salience for male and female students [21].

Most notably, the structural impulsivity–IGD association was stronger in males than in females. Given the exploratory nature of this comparison and the modest female subgroup size, this sex difference should be interpreted cautiously and treated as a preliminary finding pending replication. Conversely, the resilience–IGD association did not differ significantly across sex groups, suggesting that resilience may represent a broadly relevant correlate of IGD severity across both sexes.

### 4.5. Implications for Nursing Practice

A conceptual note warrants mention: impulsivity here is operationalized as a multidimensional self-report trait (S-UPPS-P) and should not be conflated with executive dysfunction as measured by neuropsychological performance tasks; these constructs overlap but are not interchangeable. These findings carry provisional implications for university mental health nursing, while acknowledging that this observational study did not evaluate any intervention. The indirect association through resilience suggests that resilience-building components may be a worthwhile target alongside impulsivity-focused approaches in future intervention trials; whether a sequenced “resilience-first” strategy produces better outcomes than simultaneous or impulsivity-first approaches remains a hypothesis requiring randomized or longitudinal evidence. Regarding the sex-based findings, the partial scalar non-invariance identified for two items warrants attention in future measurement work; however, the present data do not provide criterion-validity evidence sufficient to justify recommending sex-specific clinical thresholds at this stage.

### 4.6. Limitations and Future Directions

Despite its strengths, the study has several limitations. First, the cross-sectional design precludes establishing temporal ordering; reverse and reciprocal relationships are plausible (e.g., IGD symptoms reducing perceived resilience, or shared psychological distress driving all three constructs), and longitudinal panel SEM is needed before directional conclusions can be drawn. Alternative structural models were not formally tested as sensitivity analyses, which future work should address. Second, all measures were self-reported and collected simultaneously, introducing risk of common-method variance and social-desirability bias, particularly for gaming-related items. Third, eligibility was not restricted to active gamers, and gaming frequency and duration were not measured; IGDS9-SF scores therefore reflect symptom endorsement on a screening instrument rather than a confirmed clinical condition, and floor effects cannot be excluded. Fourth, the sample was drawn from a single university using cluster convenience sampling; demographic comparison with national enrollment data was not possible, limiting assessment of external validity beyond King Saud University students. Fifth, the female subgroup (*n* = 85) constrains precision of sex-specific path estimates and invariance testing; estimated power for detecting a medium path difference was approximately 0.49, meaning the sex-comparison results require replication in adequately powered samples. Sixth, the S-UPPS-P parceling strategy, while sample-size-driven, prevents examination of facet-level differential associations; studies with *n* ≥ 400 should model the five facets separately or test bifactor specifications. Seventh, important potential confounders, including depression, academic stress, psychological distress, age, gaming duration, and socioeconomic characteristics, were not included in the model, and omitted-variable bias cannot be excluded. Finally, the single-university setting and Saudi Arabian cultural context limit generalizability to other populations, and independent replication in Arab and non-Arab samples is needed.

## 5. Conclusions

This study found that the association between impulsivity and Internet Gaming Disorder (IGD) among Saudi university students was partially accounted for by lower psychological resilience as a statistical mediator in a cross-sectional latent-variable SEM. The covariance structure is consistent with resilience occupying a mediating position in the impulsivity–IGD association, though the cross-sectional design does not permit causal inference, and reverse or reciprocal relationships cannot be excluded. The results also indicated that the impulsivity–IGD association was stronger in males than in females, though this sex difference should be treated as preliminary given the modest female subgroup size and the exploratory nature of the comparison.

These results are consistent with the I-PACE model’s proposition that predisposing individual-difference variables interact with affective–cognitive regulatory processes in behavioral addiction, contributing a GCC-specific SEM dataset to this evidence base. A path diagram with standardized coefficients, residual variances, and R^2^ values is provided as Supplementary Figure S1. Future longitudinal and interventional research is needed to establish causal ordering and test whether targeting resilience alongside impulsivity improves clinical outcomes.

## Figures and Tables

**Table 1:** Confirmatory factor analysis fit indices for measurement and structural models.

Model	CFI	TLI	RMSEA	90% CI	SRMR	ΔSBS-χ^2^
Measurement model (3-factor)	0.934	0.912	0.057	[0.044, 0.069]	0.063	Reference
2-factor (IGD + Impulsivity vs. Resilience)	0.871	0.843	0.079	[0.067, 0.091]	0.081	87.4 *** (df = 3)
Structural model (full paths)	0.928	0.906	0.059	[0.046, 0.072]	0.068	3.1 (df = 1), ns

Notes: *** *p* < 0.001. Abbreviations: CFI, Comparative Fit Index; TLI, Tucker–Lewis Index; RMSEA, Root Mean Square Error of Approximation; SRMR, Standardized Root Mean Square Residual; ΔSBS-χ^2^, Satorra–Bentler scaled chi-square difference test; df, degrees of freedom; ns, not significant.

**Table 2:** Standardized structural path coefficients from the final SEM model.

Path	β	SE	z	*p*	95% CI LL	95% CI UL
Impulsivity → IGD Severity	0.38	0.08	4.75	<0.001	0.224	0.536
Resilience → IGD Severity	−0.26	0.07	−3.71	<0.001	−0.397	−0.123
Impulsivity → Resilience	−0.32	0.09	−3.56	<0.001	−0.496	−0.144
Indirect: Impulsivity → Resilience → IGD	0.083	0.030	2.77	0.006	0.031	0.149

Note: β = standardized coefficient. SE and z correspond to the unstandardized solution; 95% CIs are bias-corrected bootstrap intervals for the indirect effect and asymptotic intervals for direct effects. LL, lower limit; UL, upper limit.

**Table 3:** Multi-group measurement invariance test results across sex.

Model	CFI	RMSEA	ΔCFI	ΔRMSEA	SBS-χ^2^	df	Decision
Configural	0.919	0.063	-	-	-	-	Accept
Metric (equal loadings)	0.914	0.061	0.005	0.002	7.2	12	Accept
Scalar (equal intercepts)	0.898	0.068	0.016	0.007	24.8	12	Partial
Partial Scalar (2 intercepts freed)	0.907	0.064	0.007	0.003	14.1	10	Accept

Note: Decision based on ΔCFI ≤ 0.010 and ΔRMSEA ≤ 0.015 criteria [29].

**Table 4:** Structural Path Coefficients by Sex Under Partial Scalar Invariance Model.

Path	Males β	*p*	Females β	*p*	Path Diff. Test	Decision
Impulsivity → IGD	0.46	<0.001	0.27	0.009	Δχ^2^(1) = 4.65, *p* = 0.031	Significant
Resilience → IGD	−0.29	<0.001	−0.24	0.003	Δχ^2^(1) = 0.68, ns	Invariant
Impulsivity → Resilience	−0.35		−0.28	0.003	Δχ^2^(1) = 1.02, ns	Invariant

Note: *p* < 0.05. Unstandardized coefficients (b) and standard errors (SE) by sex: Impulsivity → IGD: males b = 0.61, SE = 0.13; females b = 0.36, SE = 0.14. Resilience → IGD: males b = −0.38, SE = 0.10; females b = −0.31, SE = 0.11. Impulsivity → Resilience: males b = −0.43, SE = 0.12; females b = −0.35, SE = 0.13. Path-difference tests used Satorra–Bentler robust Δχ^2^(1) under the partial scalar invariance model. β = standardized coefficient.

## Data Availability

The data presented in this study are available on reasonable request from the corresponding author. Data are not publicly available due to ethical restrictions on participant confidentiality.

## Supplementary Materials

The following supporting information can be downloaded at: https://www.mdpi.com/article/10.3390/healthcare14131955/s1. Supplementary File S1: STROBE Checklist (Strengthening the Reporting of Observational Studies in Epidemiology). Supplementary Figure S1: Path diagram of the final SEM with standardized coefficients, residual variances, and R^2^ values (*n* = 207; MLR estimator). Supplementary Table S1: Complete item-level CFA factor loadings (standardized and unstandardized) with 95% CIs and residual variances. Supplementary Table S2: Unstandardized direct, indirect, and total effects with bootstrapped 95% CIs.

## Abbreviations

The following abbreviations are used in this manuscript:

CFI	Comparative Fit Index
TLI	Tucker–Lewis Index
RMSEA	Root Mean Square Error of Approximation
SRMR	Standardized Root Mean Square Residual
IGD	Internet Gaming Disorder
