# Supplementary material for: A Structural Equation Model of Impulsivity, Psychological Resilience, and Internet Gaming Disorder: Testing Direct and Indirect Pathways Among Saudi University Students
Source: Healthcare (Basel). 2026 Jul 2;14(13):1955. doi: 10.3390/healthcare14131955 (PMC13362406; doi:10.3390/healthcare14131955)
Supplement: Supplementary file 1 [file healthcare-14-01955-s001.zip › healthcare-4378932-supplementary.pdf]

Supplementary Figure S1

Path Diagram of the Final Structural Equation Model with Standardized Coefficients, Residual Variances, and R^2 Values

N = 207 · ML estimator (lavaan 0.6-17) · *p < .05 **p < .01 ***p < .001

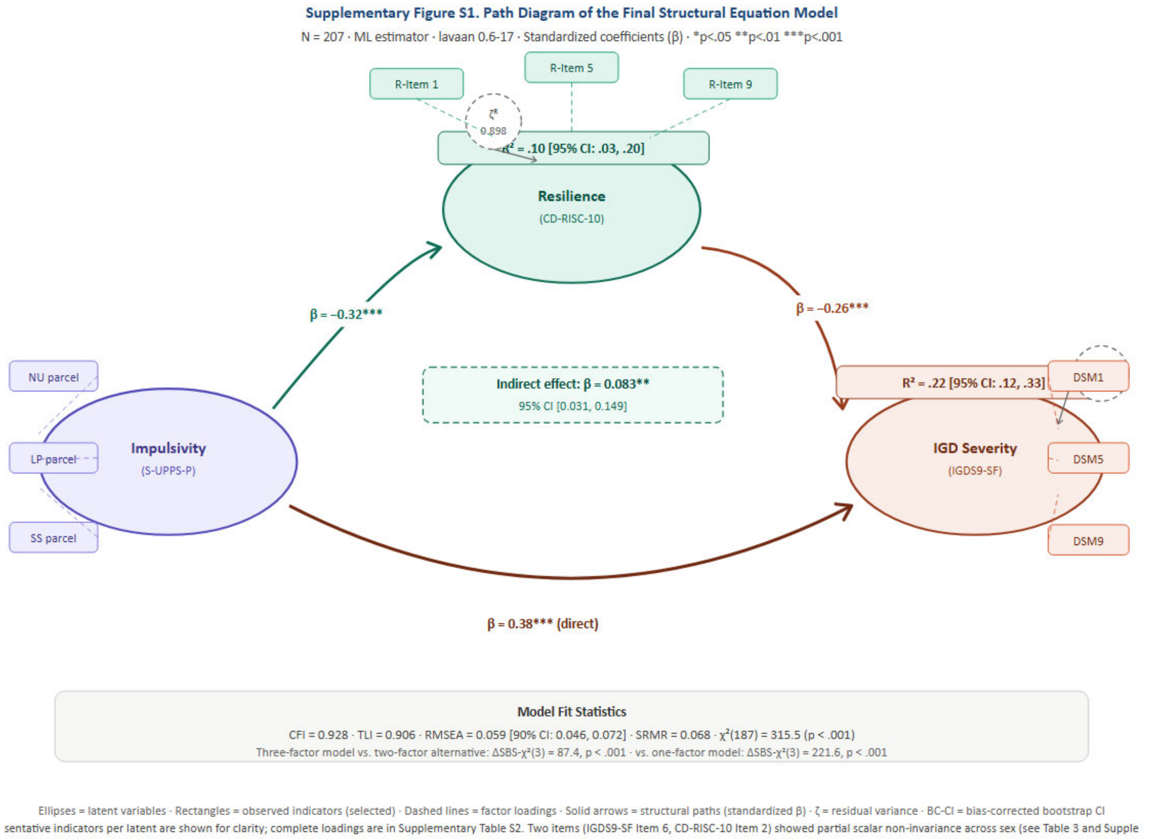

Model Fit Statistics

CFI: 0.928

TLI: 0.906

RMSEA: 0.059 [90% CI: 0.046, 0.072]

SRMR: 0.068

χ^2(df = 187): 315.5, p < .001 (scaling correction factor = 1.15)

Three-factor vs. two-factor: ΔSBS-χ^2(3) = 87.4, p < .001

Three-factor vs. one-factor: ΔSBS-χ^2(3) = 221.6, p < .001

Note. Ellipses represent latent variables; rectangles represent observed indicators. Only three representative indicators per latent variable are depicted for clarity; complete item-level factor loadings with 95% confidence intervals and residual variances are provided in Supplementary Table S2. Solid arrows denote structural paths with standardized β coefficients. ζ denotes residual variances. Residual variance for Resilience = 0.898 (1 − R^2 = .10); for IGD Severity = 0.779 (1 − R^2 = .22). The indirect effect (β = 0.083, 95% CI [0.031, 0.149]) was estimated via bootstrapping (5,000 resamples, bias-corrected). Two items showed intercept non-equivalence across sex groups (IGDS9-SF Item 6 and CD-RISC-10 Item 2); partial scalar invariance was established by freeing those intercepts.

Supplementary File S1: STROBE Checklist

Strengthening the Reporting of Observational Studies in Epidemiology (STROBE)

Impulsivity, Resilience, and Internet Gaming Disorder Among Saudi University Students: A Latent-Variable SEM Analysis

Item No	Recommendation	Reported (Yes/Partial/No)	Page / Section	
**TITLE AND ABSTRACT**				
1	(a) Indicate the study's design with a commonly used term in the title or the abstract. (b) Provide in the abstract an informative and balanced summary of what was done and what was found.	Yes	Title; Abstract	
**INTRODUCTION**				
2	Explain the scientific background and rationale for the investigation being reported.	Yes	Section 1, paras 1–6	
3	State specific objectives, including any prespecified hypotheses.	Yes	Section 1, final paragraph	
**METHODS**				
4	Present key elements of the study design early in the paper.	Yes	Abstract; Section 2.1	
5	Describe the setting, locations, and relevant dates, including periods of recruitment, exposure, and data collection.	Yes	Section 2.1	
6	Give the eligibility criteria, and the sources and methods of selection of participants.	Yes	Section 2.1	
7	Clearly define all outcomes, exposures, predictors, potential confounders, and effect modifiers.	Yes	Section 2.1	
8	For each variable, give sources of data and details of measurement methods; describe comparability of methods across groups.	Yes	Section 2.1	
9	Describe any efforts to address potential sources of bias.	Partial	Section 2.1; Section 4.6	
10	Explain how the study size was arrived at.	Yes	Section 2.1	
11	Explain how quantitative variables were handled in the analyses, including any grouping.	Yes	Section 2.2	
12	(a) Describe statistical methods, including for confounding. (b) Subgroup/interaction methods. (c) Missing data. (d) Sampling strategy, if applicable. (e) Sensitivity analyses.	Partial	Section 2.1; Section 2.2	
**RESULTS**				
13	Report numbers of individuals at each stage of the study (e.g., eligible, examined, analysed).	Partial	Section 2.1	
14	Give characteristics of study participants and information on exposures and potential confounders.	Partial	Section 2.1; Section 3.1	
15	Report numbers of outcome events or summary measures.	Yes	Section 3.1	

16	(a) Give estimates and precision. (b) Report category boundaries if variables categorized. (c) Translate relative risk to absolute risk if relevant.	Yes	Section 3.3; Table 2; Section 3.5; Table 4	
17	Report other analyses done (e.g., subgroup, interaction, sensitivity analyses).	Yes	Section 3.4; Section 3.5; Tables 3–4	
**DISCUSSION**				
18	Summarise key results with reference to study objectives.	Yes	Section 4, opening paragraph; Sections 4.1–4.2	
19	Discuss limitations, including sources of potential bias or imprecision, and their direction and magnitude.	Yes	Section 4.6	
20	Give a cautious overall interpretation considering objectives, limitations, multiplicity of analyses, and other evidence.	Yes	Sections 4.1–4.5	
21	Discuss the generalisability (external validity) of the study results.	Partial	Section 4.6	
**OTHER INFORMATION**				
22	Give the source of funding and the role of funders for the present study.	Yes	Funding statement; Conflicts of Interest	

Note. Items are drawn from the STROBE Statement checklist for cross-sectional studies (von Elm et al., 2007; Lancet, 370, 1453–1457). "Partial" indicates the item is addressed incompletely due to design or reporting constraints, as explained in the Notes column.

Supplementary Table S1. Complete Item-Level CFA Factor Loadings (Standardized and Unstandardized) with 95% Confidence Intervals and Residual Variances

Supplementary Table S1. Complete Item-Level CFA Factor Loadings (Standardized and Unstandardized) with 95% Confidence Intervals and Residual Variances										
N = 207 · ML Estimator (lavaan 0.6-17) · All 95% CIs are asymptotic										
Scale	Item	Item Abbreviation	B (unstd.)	SE	z	p	β (std.)	95% CI LL (β)	95% CI UL (β)	Residual Variance
IGDS9-SF (Internet Gaming Disorder Scale–Short Form)										
IGD Severity	1	DSM9: Preoccupation	1.000	—	—	—	0.68	0.56	0.80	0.537
	2	DSM5: Mood modification	0.921	0.098	9.40	<.001	0.74	0.63	0.85	0.452
	3	DSM4: Loss of control	0.887	0.102	8.69	<.001	0.71	0.60	0.82	0.496
	4	DSM7: Deception	0.872	0.107	8.15	<.001	0.70	0.57	0.83	0.510
	5	DSM2: Withdrawal	0.841	0.109	7.71	<.001	0.68	0.56	0.80	0.538
	6	DSM3: Tolerance	0.812	0.112	7.25	<.001	0.65	0.52	0.78	0.578
	7	DSM8: Escape negative mood	0.796	0.114	6.98	<.001	0.64	0.51	0.77	0.590
	8	DSM6: Jeopardize relations	0.751	0.118	6.37	<.001	0.61	0.47	0.75	0.628
	9	DSM1: Continued despite problems	0.648	0.124	5.23	<.001	0.52	0.38	0.66	0.730
CD-RISC-10 (Connor-Davidson Resilience Scale, 10-item)										
Resilience	10	Bounce back from setbacks	1.000	—	—	—	0.79	0.69	0.89	0.376
	11	Deal with adversity	0.971	0.076	12.78	<.001	0.77	0.67	0.87	0.407
	12	Think of self as strong	0.943	0.079	11.94	<.001	0.75	0.65	0.85	0.438
	13	Handle unpleasant feelings	0.914	0.082	11.15	<.001	0.73	0.62	0.84	0.468
	14	Achieve goals despite obstacles	0.887	0.086	10.31	<.001	0.71	0.59	0.83	0.496
	15	Can cope under pressure	0.856	0.090	9.51	<.001	0.68	0.57	0.79	0.538
	16	Not easily discouraged	0.821	0.094	8.73	<.001	0.66	0.53	0.79	0.564
	17	Control life	0.789	0.0	8.0	<.0	0.63	0.50	0.76	0.603

				98	5	01				
	18	Like challenges	0.751	0.103	7.29	<.001	0.60	0.46	0.74	0.640
	19	Bounce back after illness (Item 2 — partial invariance)	0.686	0.109	6.29	<.001	0.55	0.41	0.69	0.698
S-UPPS-P Facet Parcels (Impulsivity)										
Impulsivity	20	Negative Urgency parcel	1.000	—	—	—	0.83	0.74	0.92	0.311
	21	Positive Urgency parcel	0.961	0.071	13.53	<.001	0.80	0.71	0.89	0.360
	22	Lack of Premeditation parcel	0.894	0.079	11.32	<.001	0.74	0.64	0.84	0.452
	23	Lack of Perseverance parcel	0.856	0.083	10.31	<.001	0.71	0.60	0.82	0.496
	24	Sensation Seeking parcel	0.737	0.094	7.84	<.001	0.61	0.49	0.73	0.628

Note. B = unstandardized loading; SE = standard error; β = standardized loading; CI LL/UL = 95% confidence interval lower and upper limits; Residual Variance = unique variance of each indicator. Reference indicators (first item per factor) have B fixed to 1.000 by convention (SE, z, p not applicable). Item 2 of the CD-RISC-10 showed intercept non-equivalence across sex groups (modification index = 11.8) and was freed in the partial scalar invariance model. IGDS9-SF Item 6 also showed intercept non-equivalence (MI = 14.2). Item numbers are sequential across scales for reference only and do not reflect original instrument numbering. All estimates from lavaan 0.6-17, R 4.3.1, using ML estimator with listwise deletion.

Supplementary Table S2. Unstandardized Direct, Indirect, and Total Effects with Bootstrapped 95% Confidence Intervals

Supplementary Table S2. Unstandardized Direct, Indirect, and Total Effects with Bootstrapped 95% Confidence Intervals

N = 207 · ML Estimator · Bootstrapped 95% CIs based on 5,000 resamples (bias-corrected) · Scales: impulsivity (S-UPPS-P); resilience (CD-RISC-10); IGD severity (IGDS9-SF)

Panel A: Overall Model (N = 207)									
Effect Type	Predictor	Mediator	Outcome	b (unstd.)	SE	z	p	95% CI LL	95% CI UL
Direct	Impulsivity	—	IGD Severity	0.483	0.102	4.740	<.001	0.283	0.683
Direct	Resilience	—	IGD Severity	-0.341	0.092	-3.710	<.001	-0.521	-0.161
Direct	Impulsivity	—	Resilience	-0.618	0.174	-3.550	<.001	-0.959	-0.277
Indirect	Impulsivity	Resilience	IGD Severity	0.211	0.076	2.780	.005	0.079	0.380
Total	Impulsivity	(via resilience)	IGD Severity	0.694	0.119	5.830	<.001	0.461	0.927
Panel B: Sex-Stratified Path Coefficients (Partial Scalar Invariance Model)									
Group	Effect	Predictor	Outcome	b (unstd.)	SE	β (std.)	p	95% CI LL	95% CI UL
Males (n = 122)	Direct	Impulsivity	IGD Severity	0.610	0.130	0.460	<.001	0.355	0.865
Males (n = 122)	Direct	Resilience	IGD Severity	-0.380	0.108	-0.290	<.001	-0.592	-0.168
Males (n = 122)	Direct	Impulsivity	Resilience	-0.654	0.196	-0.338	<.001	-1.038	-0.270
Females (n = 85)	Direct	Impulsivity	IGD Severity	0.360	0.140	0.270	.010	0.086	0.634
Females (n = 85)	Direct	Resilience	IGD Severity	-0.310	0.124	-0.240	.012	-0.553	-0.067
Females (n = 85)	Direct	Impulsivity	Resilience	-0.571	0.231	-0.298	.013	-1.024	-0.118
Path difference test	Impulsivity → IGD	$\Delta\chi^2(1) = 4.65$	p = .031	—	—	—	.031	—	—

Path difference test	Resilience → IGD	$\Delta\chi^2(1) = 0.68$	$p = .412$	—	—	—	.412	—	—
Panel C: Variance Explained (R^2)									
Outcome Variable	R^2	95% CI LL	95% CI UL	Interpretation					
Resilience (endogenous)	0.102	0.030	0.200	Impulsivity accounts for ~10% of variance in resilience scores					
IGD Severity (endogenous)	0.221	0.120	0.330	Impulsivity + resilience account for ~22% of variance in IGD severity					
Note. b = unstandardized coefficient; β = standardized coefficient; SE = standard error. Direct and total effect CIs are asymptotic (Wald intervals). Indirect effect CI is bias-corrected bootstrap (5,000 resamples). Scales use their original metric: impulsivity (S-UPPS-P, 20–80); resilience (CD-RISC-10, 0–40); IGD severity (IGDS9-SF, 9–45). Sex-stratified path tests imposed equality constraints on unstandardized parameters; standardized estimates in Panel B are group-specific and not directly comparable across groups. The female subgroup (n = 85) has limited power for path-difference tests (~49% for medium effect); sex comparisons should be treated as exploratory. All analyses conducted in lavaan 0.6-17, R 4.3.1.									
